# Resident-side health information for identifying community noise risks in digital public health governance

**DOI:** 10.3389/fpubh.2026.1926467

**Published:** 2026-08-03

**Authors:** Yi Jiang, Bin Dai, Zhi Mo

**Affiliations:** 1 Law School, Peking University, Beijing, China; 2 Shanghai University of Engineering Science, Shanghai, China; 3 Law School, Shanghai University of Finance and Economics, Shanghai, China

**Keywords:** community noise exposure, digital public health governance, public health risk identification, resident-reported health information, sleep disturbance, source-specific governance

## Abstract

**Introduction:**

Digital public health governance needs to identify community-level health risks beyond data generated after residents enter medical-service settings. In this study, resident-side health information refers to resident-reported information on residential noise perception, sleep disturbance, and health behavior that can supplement medical-service data. Noise pollution is a frequently perceived environmental problem in everyday life, and China’s source-based noise governance framework needs resident-side evidence on whether different noise sources correspond to different sleep-related risks.

**Methods:**

This study used cross-sectional questionnaire data from urban residents in China. A total of 451 valid responses were retained. Noise exposure was measured through subjective noise annoyance and self-reported residential noise-environment proxy indicators. Sleep disturbance and health behavior were also measured through self-reported items. The analysis included descriptive statistics, reliability and validity tests, correlation analysis, regression models, mediation analysis, and exploratory comparisons across source-specific noise annoyance.

**Results:**

Subjective noise annoyance (*β* = 0.427, *p* < 0.001), self-reported residential noise-environment proxy indicators (*β* = 0.466, *p* < 0.001), and overall noise exposure (*β* = 0.668, p < 0.001) were positively associated with sleep disturbance. Sleep disturbance was negatively associated with health behavior (*β* = −0.443, *p* < 0.001). The indirect statistical pathway from noise exposure to health behavior through sleep disturbance was significant (effect = −0.296, bootstrap 95% CI [−0.375, −0.222]). Source-specific comparisons showed more stable associations for construction and renovation noise, traffic noise, and social-life noise, whereas industrial noise did not show a stable association in the present sample.

**Discussion:**

In this cross-sectional self-reported sample, community noise exposure was linked to health behavior through sleep disturbance as a statistical pathway rather than as causal evidence. The findings provide exploratory resident-side evidence for identifying community noise risks and suggest that source-specific noise governance should consider how different noise sources enter residents’ rest and recovery contexts.

## 1 Introduction

Medical-service, disease-diagnosis, and care-seeking data provide important bases for identifying public health risks (1–5). These data, however, usually record health conditions after residents have already entered medical-service settings. Some latent risks may therefore have already become clinically visible health problems by the time they are captured. A governance approach that relies only on outcome-oriented medical data can miss community-level risks that have not yet entered the medical system but have already affected daily life. Non-medical-service data, including environmental complaints, residential experience, and everyday behavior, may capture health disturbances and risk factors that remain upstream of clinical diagnosis. Noise pollution is a clear example. In the 2025 national ecological and environmental petition and complaint platform managed by the Ministry of Ecology and Environment of China, noise complaints accounted for 54.0% of all public environmental complaints and ranked first among environmental pollution factors (6). This high share indicates that noise has become an environmental problem that residents frequently perceive and actively report in everyday life, and it points to the need to examine whether community noise is associated with sleep disturbance, changes in daily rhythms, and health behavior (7–12).

The association between noise exposure and multiple health outcomes has a substantial evidence base. Guidance from the World Health Organization indicates that excessive noise can increase risks related to sleep disturbance, annoyance, ischemic heart disease, hypertension, hearing impairment, tinnitus, and cognitive impairment in children (13). For community residents, these risks may first appear as disrupted rest at night, increased subjective annoyance, and forced adjustments to daily routines. Sleep disturbance is therefore an important entry point for observing the relationship between community noise exposure and residents’ health status. Daily health behavior may also be related to inadequate sleep recovery and disrupted life rhythms. Existing studies have separately examined noise exposure and sleep disturbance, and sleep disturbance and health behavior, but fewer studies have placed the three into one empirical framework. Community noise also does not come from one source. Traffic, construction and renovation, industrial activity, and social life enter residents’ daily life at different times and in different contexts, and they differ in perceived controllability. Chinese noise pollution law and the ecological environmental code both adopt source-based governance for industrial, construction, traffic, and social-life noise. If risk is measured only by overall noise exposure, differences between noise sources and sleep-related disturbance may be obscured.

Whether this source-based governance framework responds to noise-related interference with rest and sleep still lacks empirical evidence from resident-side health information. Here, resident-side health information means resident-reported information on perceived residential noise exposure, sleep disturbance, and daily health behavior that is collected before or outside medical-service encounters. This study compares traffic, construction and renovation, industrial, and social-life noise within the same community context to examine whether the legal classification corresponds to observable differences in sleep-related health risk. The legal recognition of noise pollution is the starting point of noise prevention and control, and it also informs regulatory procedures and legal responsibility (14). On this basis, this study examines the statistical pathway from noise exposure to health behavior through sleep disturbance and compares the associations between different noise sources and sleep disturbance. The aim is to provide empirical evidence for community-level noise risk identification, source-specific governance under China’s ecological environmental code framework, and the use of resident-side data in digital public health governance. It also clarifies how sleep disturbance may serve as resident-side evidence for debates about rest and sleep tranquility, regulatory classification, and possible remedies, without treating cross-sectional survey associations as legal proof of causation.

## 2 Literature review

Environmental noise has been linked to multiple health outcomes, but the analytical focus of this study is narrower. It asks how noise disturbance perceived by community residents in residential environments first appears as sleep disturbance and then relates to daily health behavior. Around this main line, the literature needs to clarify why sleep disturbance is a suitable proximal indicator of community noise risk, why different noise sources should be distinguished within the same community context, and why sleep disturbance may connect environmental exposure with health behavior.

These three issues also correspond to three practical questions: the identification of health harm, the empirical basis for source-specific governance, and the evidentiary pathway for linking environmental disturbance with downstream behavior. Sleep disturbance responds to the legal and policy question of how harm can be observed. The Chinese Noise Pollution Prevention and Control Law defines noise pollution through both emission standards and interference with normal life, work, and study. Because interference with normal life is a broad normative expression, sleep disturbance provides a more observable resident-side indicator that can connect administrative control with health-oriented governance. Only when the evidence is placed within one analytical chain can resident-side health information show how community noise risk becomes empirically identifiable.

### 2.1 Community noise exposure, source differences, and sleep disturbance

Sleep disturbance is one of the most stable proximal health indicators in noise research and is well suited to community life contexts (8, 13). Existing studies commonly distinguish auditory from non-auditory effects of environmental noise (7). Non-auditory effects include distal outcomes such as cardiovascular disease and cognitive impairment (15–17), as well as more immediate experiences such as annoyance, sleep disruption, and reduced quality of life (7, 8, 18, 19). Compared with distal outcomes, proximal effects better reflect how external environmental disturbance affects individual recovery (8, 20), indicate potential health risks (13, 15), and can be perceived and reported by residents in daily life.

The strongest evidence on noise and sleep has accumulated in traffic-noise research (8, 21–26). Road, railway, and aircraft noise usually have relatively clear source boundaries and measurable exposure indicators (27–29), which makes them easier to incorporate into noise maps, nighttime sound levels, and event-count measures (28, 29). Systematic reviews and population studies using these indicators show statistical associations between nighttime traffic noise and self-reported sleep disturbance or sleep medication use (8, 22, 25). Experimental and physiological studies further suggest that some noise sources may affect sleep processes through heart rate, electroencephalographic changes, or stress-hormone responses even when they do not cause full awakening (30–34). This evidence supports the use of sleep disturbance as a proximal indicator of noise-related health risk. Yet it remains concentrated mainly on traffic sources, while community residents often encounter multiple sources that enter rest contexts in different ways.

Different noise sources should not be collapsed into one overall exposure without qualification (13, 35). Their relevance for sleep disturbance depends on timing, intensity, predictability, controllability, and the route through which noise enters residential space (23, 35). Source type corresponds not only to governance categories (6, 13), but also to different temporal patterns and residential contexts (28, 29). Traffic noise may have stable background levels and nighttime events (22–26, 35). Construction and renovation noise may involve episodic high-intensity disturbance and overlap with daytime or rest periods (36, 37). Social-life noise is embedded in neighborhood, commercial, entertainment, and public activity settings and may carry stronger social and perceived-controllability features (38). Industrial noise may depend more on the spatial distance between residences and sources, functional zoning, and the exposure distribution within the sample (6, 13). Noise-annoyance research also shows that judgments about whether a source is predictable, avoidable, or controllable can shape reactions to the same acoustic stimulus (15, 18, 19). Source differences are therefore not merely an additional classification outside overall noise exposure; they are an empirical dimension for understanding community noise risks.

Existing research already shows that noise exposure is associated with sleep disturbance, but two questions remain insufficiently answered in multi-source community contexts. First, traffic-noise studies often rely on objective sound levels, noise maps, or specific traffic-source exposure, while ordinary residents’ subjective perception of multiple sources in residential environments is less often examined. Second, sleep problems are usually treated as health outcomes of noise exposure, while fewer studies examine whether sleep problems are also associated with residents’ daily health behavior. For risk identification based on resident-side health information, it is not enough to confirm an association between noise and sleep. It is also necessary to examine whether this association indicates changes at the level of health behavior.

### 2.2 Sleep disturbance, health behavior, and the integration gap

Research on sleep and health behavior has more often examined how health behavior improves sleep (39, 40). This pathway provides a basis for understanding the relationship between activity and sleep, but it also limits its usefulness for the present question. Exercise intervention and health-behavior studies usually treat health behavior as a behavioral resource that promotes nighttime recovery, emphasizing the role of regular activity in improving sleep quality, shortening sleep latency, or relieving insomnia symptoms (41). With diaries, accelerometers, and wearable devices increasingly used in health-behavior research, sleep and activity have been examined at finer temporal scales. The research perspective has expanded from the effect of activity on sleep to dynamic relationships between sleep status and subsequent activity levels, sedentary behavior, and moderate-to-vigorous physical activity (42–45). This evidence indicates a dynamic association between sleep and health behavior.

Sleep reflects individual recovery status and also shapes daytime energy, life rhythm, and the ability to maintain behavior. It can therefore serve as a connecting factor between environmental noise exposure and health behavior (11, 12, 42–44, 46). Health behavior requires time arrangement, physical recovery, motivation, and self-regulatory capacity. Insufficient or poor-quality sleep may weaken these conditions (46–48). For community residents exposed to persistent noise disturbance, sleep disturbance may appear as daytime fatigue, reduced attention, and lower willingness to be active, and may be associated with walking, exercise, and moderate-to-vigorous activities that require active effort (49). Existing sleep and health-behavior studies provide empirical support for this association, but most do not include environmental noise as an upstream exposure. They therefore cannot show whether noise-related sleep disturbance is also linked to health behavior.

The current literature has not fully revealed the relationship between multi-source noise and residents’ daily health behavior. It provides two relatively clear evidence chains: one between noise exposure and sleep disturbance, and another between sleep disturbance and health behavior. Noise-health studies have examined the associations between environmental noise, annoyance, sleep disturbance, and some distal outcomes. Sleep and health-behavior studies have shown that sleep status may affect the energy, rhythm, and self-regulation required to maintain daily activity. Yet noise research often treats sleep disturbance as the endpoint, health-behavior research often separates activity from environmental exposure, and source-difference research remains concentrated on traffic noise (28). This study therefore asks two questions: Q1, is noise exposure associated with health behavior through sleep disturbance? Q2, do associations differ across noise sources?

Answering these questions can provide resident-side evidence for identifying community noise health risks. Environmental tort and regulatory settings often face difficulty when linking noise exposure to distal health outcomes such as cardiovascular disease or cognitive impairment. A proximal indicator such as sleep disturbance may make the association more observable, although cross-sectional survey evidence cannot establish strict legal causality. This study therefore places sleep disturbance between noise exposure and health behavior and compares source-specific associations as an empirical supplement to community noise risk identification.

## 3 Methods

To answer these questions, this study translated community noise exposure, sleep disturbance, and health behavior into measurable variables. The methodological task was to define the conceptual boundaries, model roles, and measurement scope of these variables so that the noise exposure, sleep disturbance, and health behavior relationship identified in the literature could be examined statistically.

The study used questionnaire data from urban residents. A total of 493 questionnaires were collected through an online survey platform. After screening by response time and an attention-check item, 451 valid questionnaires were retained, yielding an effective response rate of 91.4%. Because the data are cross-sectional and self-reported, all results are interpreted as statistical associations and statistical pathway evidence observed at the same or recent time point, not as strict temporal causality, exposure effects, or intervention effects.

### 3.1 Operational definitions of variables

The study focused on three core variables: noise exposure, sleep disturbance, and health behavior. Noise exposure refers to noise disturbance perceived by residents in residential environments and daily life contexts. This variable was divided into two subdimensions: subjective noise annoyance and self-reported residential noise-environment proxy indicators. This design distinguishes residents’ noise reactions from their reports of environmental conditions and allows the analysis to examine whether community noise information is statistically associated with sleep disturbance and health behavior.

Sleep disturbance refers to sleep problems that residents can perceive and report in daily life. In the research model, sleep disturbance is treated as a mediator for examining whether community noise exposure is associated with health behavior through disturbed recovery status.

Health behavior refers to physical activity behaviors in daily life, work, travel, and leisure contexts. The study treats health behavior as the outcome variable and examines its statistical relationship with noise exposure and sleep disturbance.

### 3.2 Hypotheses and exploratory question

From an environmental-stress perspective, community noise can be understood as a continuous or intermittent environmental stressor. Existing noise-health research indicates that noise exposure may activate physiological stress responses, including hypothalamic–pituitary–adrenal axis activation and sympathetic arousal, and may thereby disturb normal sleep rhythms through difficulty falling asleep, sleep interruption, and reduced deep sleep (27, 33). For community residents, noise entering residential and rest contexts may affect recovery by disturbing sleep onset, sleep continuity, sleep duration, and daytime fatigue. This study therefore proposes H1: community noise exposure is positively associated with residents’ sleep disturbance.

Within noise-exposure measurement, subjective noise annoyance and self-reported residential noise-environment proxy indicators correspond to different information sources. Subjective annoyance emphasizes psychological response and perceived disturbance, while the proxy indicators emphasize residents’ reports of residential noise conditions. Both are resident-side noise information, but they capture different angles. This study further proposes H1a: subjective noise annoyance is positively associated with sleep disturbance, and H1b: self-reported residential noise-environment proxy indicators are positively associated with sleep disturbance.

Sleep disturbance may further relate to residents’ ability to maintain health behavior. Sleep disruption can increase daytime fatigue and reduce energy. It may also weaken motivation and self-regulation needed for daily activity, exercise, and moderate-to-vigorous activity. Based on the relationship between sleep recovery and maintenance of health behavior, this study proposes H2: sleep disturbance is negatively associated with health behavior.

H1 and H2 together imply a connecting role for sleep disturbance between community noise exposure and health behavior. Noise exposure may first be associated with disturbed sleep, and inadequate recovery may then be associated with lower health behavior scores. Sleep disturbance can therefore be understood as a statistical pathway through which environmental exposure is linked to health behavior (48, 49). This study proposes H3: sleep disturbance mediates the statistical association between community noise exposure and health behavior.

**Figure 1:**
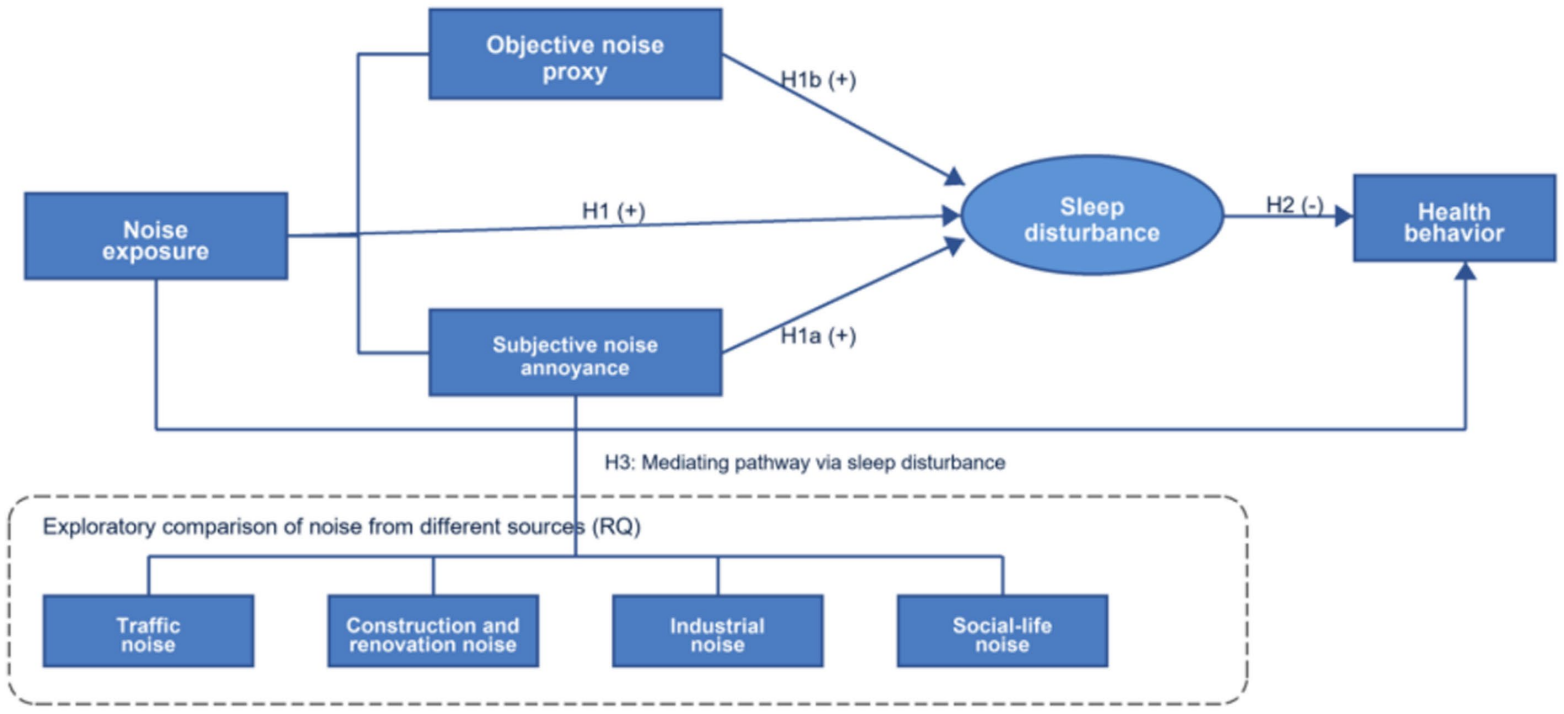
Research model linking resident-side health information to community noise risk identification.

Community noise also differs by source. Traffic, construction and renovation, industrial, and social-life noise differ in timing, continuity, predictability, controllability, and routes into residential space (26, 28, 36–38). Because the evidence base for different sources is uneven, this study does not set directional hypotheses about which source has the strongest association. It instead asks the exploratory question: RQ, do associations between source-specific noise annoyance and sleep disturbance differ across noise sources? (see Figure 1).

### 3.3 Measurement and questionnaire items

The questionnaire collected resident-side health information on residential noise perception, sleep disturbance, health behavior, and demographic characteristics.

The noise-exposure section referred to environmental noise response and noise-annoyance measures by including an overall noise-annoyance item and scenario-based items for different noise types. It also included self-reported residential noise-environment proxy indicators, following community noise and health research and traffic-noise sleep research that consider residential environmental characteristics. The source-specific items were designed in accordance with the categories of industrial noise, construction noise, traffic noise, and social-life noise in the Noise Pollution Prevention and Control Law of the People’s Republic of China (50). Because different noise sources may appear with different frequency and contextual complexity in community settings, the source-specific annoyance items are treated as exploratory source-level indicators rather than as fully equivalent mature latent constructs.

Sleep disturbance items were adapted from the Pittsburgh Sleep Quality Index and its Chinese reliability and validity research. To reduce respondent burden and maintain questionnaire consistency, the study adapted the measure to a five-point Likert format (51, 52). Health behavior items referred to the World Health Organization physical activity guidelines, the International Physical Activity Questionnaire, and Chinese IPAQ validation research. Because the questionnaire was adapted to the present research focus and survey length, the results are interpreted as self-reported health behavior rather than the full activity volume calculated under the original IPAQ scoring system (53–56).

The questionnaire contained 40 substantive items, four demographic items on gender, age, occupation, and city type, and one attention-check item, for a total of 45 items (Table 1).

**Table 1:** Core variables and measurement content.

Variable	Dimension	Measurement content
Noise exposure	Residential noise-environment proxy	Self-reported residential noise-environment proxy characteristics
Noise exposure	Noise annoyance	Industrial noise annoyance
Noise exposure	Noise annoyance	Construction and renovation noise annoyance
Noise exposure	Noise annoyance	Traffic noise annoyance
Noise exposure	Noise annoyance	Social-life noise annoyance
Noise exposure	Noise annoyance	Overall noise annoyance
Sleep disturbance	Sleep-related disturbance	Sleep onset, sleep duration, subjective sleep quality, sleep interruption or sleep disorder, sleep medication use, daytime sleepiness, and energy status
Health behavior	Physical activity behavior	Frequency and duration of vigorous activity, moderate activity, walking, and self-rated overall activity level

All core substantive items used a five-point Likert-type response format. Higher scores indicate higher perceived noise exposure or annoyance, greater sleep disturbance, or higher self-reported physical activity behavior, respectively.

## 4 Results

The study questionnaire was distributed through an online survey platform to participants, and 493 responses were collected. After screening based on response duration and an attention-check item, 451 valid questionnaires were retained, yielding an effective response rate of 91.4%. Because this study uses cross-sectional self-reported questionnaire data, the following results are interpreted as statistical associations and statistical pathway evidence observed at the same survey time point or within a recent time interval, rather than as strict temporal causality, exposure effects, or intervention effects.

### 4.1 Descriptive statistics

The demographic distribution (Table 2) shows that the sample covered different age groups, occupational statuses, and city types. Respondents were not concentrated in one age group, occupation, or residential context, which provides a basic empirical basis for examining community noise experience and daily health behavior.

**Table 2:** Demographic characteristics of the sample.

Variable	Category	Frequency (*n*)	Percentage (%)
Gender	Male	184	40.8
	Female	267	59.2
Age	Under 18	56	12.4
	18–30	93	20.6
	31–40	99	22.0
	41–50	85	18.8
	51–60	80	17.7
	61 and above	38	8.4
Occupation	Employed	280	62.1
	Retired	97	21.5
	Student	74	16.4
City type	Municipality	49	10.9
	Provincial capital	118	26.2
	Prefecture-level city	154	34.1
	County-level city	89	19.7
	Township or rural area	41	9.1

*N* = 451. Percentages may not sum to 100 because of rounding. Blank cells in the variable column indicate continuation of the preceding demographic variable.

For the core variables, noise exposure, sleep disturbance, and health behavior all showed sufficient within-sample variation for further analysis. The mean overall noise exposure score was 3.005 (SD = 0.780, median = 3.059). The mean sleep disturbance score was 3.065 (SD = 1.314, median = 3.062), and the mean health behavior score was 2.892 (SD = 1.270, median = 2.857). Values covered the main range of the scale, indicating that respondents were not concentrated in a single low-exposure, low-disturbance, or single-activity condition. Source-specific noise annoyance also showed observable distributions. The means were 2.943 for industrial noise, 3.021 for construction and renovation noise, 2.990 for traffic noise, and 2.989 for social-life noise. These distributions support further source-specific comparisons (Table 3).

**Table 3:** Descriptive distribution of core variables.

Variable	*n*	Mean	SD	Min	Median	Max
Health behavior	451	2.892	1.270	1.000	2.857	5.000
Sleep disturbance	451	3.065	1.314	1.000	3.062	5.000
Overall noise exposure	451	3.005	0.780	1.059	3.059	4.765
Industrial noise annoyance	451	2.943	1.294	1.000	3.000	5.000
Construction and renovation noise annoyance	451	3.021	1.236	1.000	3.000	5.000
Traffic noise annoyance	451	2.990	1.231	1.000	3.000	5.000
Social-life noise annoyance	451	2.989	1.217	1.000	3.000	5.000

All variables were scored on a 1–5 scale. Higher scores indicate higher perceived noise exposure or annoyance, greater sleep disturbance, or higher self-reported physical activity behavior, respectively. SD, standard deviation.

### 4.2 Reliability and validity analysis

The study used Cronbach’s alpha to test internal consistency. The overall questionnaire alpha was 0.922. Noise exposure had an alpha of 0.862 across 17 items. The self-reported residential noise-environment proxy subdimension had an alpha of 0.896, and the subjective noise-annoyance subdimension had an alpha of 0.790. The adapted sleep disturbance and health behavior scales had alphas of 0.976 and 0.934, respectively. The alpha values for the four source-specific annoyance indicators ranged from 0.839 to 0.883. Most dimensions therefore showed good reliability. Because the industrial noise annoyance indicator contained only two items, it is interpreted as an exploratory source-specific indicator rather than as an independent mature measurement tool (Table 4).

**Table 4:** Reliability test results.

Dimension	Number of items	Cronbach’s alpha
Noise exposure	17	0.862
Residential noise-environment proxy	4	0.896
Subjective noise annoyance	13	0.790
Industrial noise annoyance	2	0.839
Construction and renovation noise annoyance	3	0.869
Traffic noise annoyance	3	0.883
Social-life noise annoyance	3	0.883
Sleep disturbance	16	0.976
Health behavior	7	0.934

Cronbach’s *α* is reported for internal consistency. Higher *α* values indicate stronger internal consistency within the corresponding dimension.

The validity analysis showed that the overall KMO value was 0.966, and Bartlett’s test of sphericity was significant (Chi-square = 15301.143, *df* = 780, *p* < 0.001). These results indicate that the data were suitable for factor analysis and had adequate structural validity. The KMO values for noise exposure, sleep disturbance, health behavior, and related modules ranged from 0.759 to 0.986, all above 0.7, and all Bartlett tests were significant at *p* < 0.001. These results support the use of composite indicators in subsequent regression and mediation models (Table 5).

**Table 5:** Validity analysis results.

Module	*n*	Items	KMO	Bartlett chi-square	*df*	*p*
Overall questionnaire items	451	40	0.966	15301.143	780	<0.001
Noise exposure	451	17	0.832	4395.131	136	<0.001
Residential noise-environment proxy	451	4	0.845	1048.283	6	<0.001
Subjective noise annoyance	451	13	0.759	3089.543	78	<0.001
Sleep disturbance	451	16	0.986	7477.162	120	<0.001
Health behavior	451	7	0.922	2356.795	21	<0.001

KMO = Kaiser–Meyer–Olkin measure of sampling adequacy; *df* = degrees of freedom. Bartlett chi-square refers to Bartlett’s test of sphericity.

### 4.3 Correlation analysis

Pearson correlation analysis was used to examine bivariate relationships among the study variables. Noise exposure was positively correlated with sleep disturbance (*r* = 0.786, *p* < 0.001) and negatively correlated with health behavior (*r* = −0.576, *p* < 0.001). Sleep disturbance was negatively correlated with health behavior (*r* = −0.671, *p* < 0.001). Both the self-reported residential noise-environment proxy and subjective noise annoyance were positively correlated with sleep disturbance, providing a basis for subsequent regression and pathway tests. Because overall noise exposure is constructed from its lower-level dimensions, correlations among noise indicators should be interpreted hierarchically rather than as independent theoretical relationships (Table 6).

**Table 6:** Correlation analysis results.

Variable	Mean	SD	1	2	3	4	5
1. Noise exposure	3.005	0.780	—				
2. Residential noise-environment proxy	3.027	1.246	0.817***	—			
3. Subjective noise annoyance	2.998	0.740	0.954***	0.607***	—		
4. Sleep disturbance	3.065	1.314	0.786***	0.613***	0.765***	—	
5. Health behavior	2.892	1.270	−0.576***	−0.477***	−0.546***	−0.671***	—

Pearson correlations are reported. Blank cells indicate the upper triangle of the correlation matrix. ***p < 0.001. Higher scores indicate higher perceived noise exposure or annoyance, greater sleep disturbance, or higher self-reported physical activity behavior.

### 4.4 Hypothesis tests

#### 4.4.1 Noise exposure and sleep disturbance

After controlling for demographic variables (Table 7), subjective noise annoyance was positively associated with sleep disturbance (*p* < 0.001, 95% CI [0.352, 0.502]). The self-reported residential noise-environment proxy was also positively associated with sleep disturbance (*p* < 0.001, 95% CI [0.392, 0.539]). H1a and H1b were supported.

**Table 7:** Regression analysis of noise exposure and sleep disturbance.

Hypothesis	Path	*R* ^2^	Adjusted *R*^2^	Effect/*β*	SE (HC3)	*t*	*p*	LLCI	ULCI	Conclusion
H1a	Subjective noise annoyance→sleep disturbance	0.509	0.485	0.427	0.038	11.100	<0.001	0.352	0.502	Supported
H1b	Residential noise-environment proxy→sleep disturbance	0.534	0.511	0.466	0.037	12.488	<0.001	0.392	0.539	Supported
H1 joint, subjective	Subjective noise annoyance→sleep disturbance	0.603	0.583	0.306	0.039	7.879	<0.001	0.230	0.382	Supported
H1 joint, objective	Residential noise-environment proxy→sleep disturbance	0.603	0.583	0.361	0.042	8.704	<0.001	0.280	0.443	Supported
H1	Noise exposure→sleep disturbance	0.666	0.660	0.668	0.030	21.753	<0.001	0.609	0.728	Supported

*N* = 451. *R*^2^ = coefficient of determination; *β* = standardized coefficient; SE, standard error; HC3 = heteroskedasticity-consistent standard error; LLCI/ULCI = lower/upper limit of the 95% confidence interval. Higher noise-exposure scores indicate higher perceived exposure or annoyance, and higher sleep disturbance scores indicate greater sleep disturbance.

When subjective noise annoyance and the self-reported residential noise-environment proxy entered the model together, both remained significantly associated with sleep disturbance. The coefficient for subjective noise annoyance remained significant (*p* < 0.001, 95% CI [0.230, 0.382]), and the coefficient for the self-reported residential noise-environment proxy also remained significant (*p* < 0.001, 95% CI [0.280, 0.443]). The model *R*^2^ increased to 0.603, with adjusted *R*^2^ = 0.583. This result indicates that subjective annoyance and residential noise-environment proxy characteristics provide complementary information for explaining sleep disturbance.

When overall noise exposure was used to test H1, the results again showed a positive association with sleep disturbance (*p* < 0.001, 95% CI [0.609, 0.728]). H1 was therefore supported.

#### 4.4.2 Sleep disturbance and health behavior

Regression analysis showed a negative association between sleep disturbance and health behavior. With sleep disturbance as the independent variable and health behavior as the dependent variable, the coefficient was negative and significant (*p* < 0.001, 95% CI [−0.555, −0.331]). After controlling for relevant variables, respondents with higher sleep disturbance scores tended to report lower health behavior scores. The model *R*^2^ was 0.527 and adjusted *R*^2^ was 0.502, indicating stable explanatory power. H2 was supported (Table 8).

**Table 8:** Regression analysis of sleep disturbance and health behavior.

Hypothesis	Path	*R* ^2^	Adjusted *R*^2^	Effect/*β*	SE (HC3)	*t*	*p*	LLCI	ULCI	Conclusion
H2	Sleep disturbance→health behavior	0.527	0.502	−0.443	0.057	−7.742	<0.001	−0.555	−0.331	Supported

*N* = 451. *R*^2^ = coefficient of determination; *β* = standardized coefficient; SE = standard error; HC3 = heteroskedasticity-consistent standard error; LLCI/ULCI = lower/upper limit of the 95% confidence interval. Higher sleep disturbance scores indicate greater sleep disturbance, and higher health behavior scores indicate higher self-reported physical activity behavior.

#### 4.4.3 Mediating role of sleep disturbance

The mediation test showed a significant statistical pathway through sleep disturbance. Before sleep disturbance entered the model, the total association between noise exposure and health behavior was significant (*p* < 0.001, 95% CI [−0.465, −0.294]). After sleep disturbance was included, the direct association between noise exposure and health behavior was no longer significant (c prime path: *p* = 0.135, 95% CI [−0.193, 0.026]). The indirect association through sleep disturbance was significant (effect = −0.296, bootstrap 95% CI [−0.375, −0.222]). In this sample, the negative association between noise exposure and health behavior can be statistically explained through sleep disturbance. H3 was supported as a statistical mediation pathway (Table 9).

**Table 9:** Mediation analysis results.

Effect	Path	Effect/*β*	SE/BootSE	*t*/*z*	*p*	BootLLCI	BootULCI	Conclusion
Total effect	Noise exposure →health behavior	−0.379	0.044	−8.694	<0.001	−0.465	−0.294	Supported
Direct effect	Noise exposure →health behavior	−0.083	0.056	−1.494	0.135	−0.193	0.026	Not significant
Indirect effect	Noise exposure →sleep disturbance→health behavior	−0.296	0.039	−7.294	<0.001	−0.375	−0.222	Supported

*N* = 451. *β*/effect values are standardized estimates. BootSE = bootstrap standard error; BootLLCI/BootULCI = lower/upper limit of the bootstrap 95% confidence interval. The mediation result is interpreted as a statistical pathway in cross-sectional self-reported data, not as causal evidence.

### 4.5 Exploratory heterogeneity across source-specific noise annoyance

To support the exploratory interpretation, the study examined source-specific associations from three angles: simple correlations, multivariable independent associations, and coefficient-difference tests. Simple correlations show the initial bivariate relationships between each noise source and sleep disturbance. The multivariable model examines whether each source remains independently associated with sleep disturbance when the four sources enter the model together. Coefficient-difference tests further examine whether the strength of association differs across sources.

Simple correlations showed that source-specific associations were not identical (Table 10). Construction and renovation noise annoyance, traffic noise annoyance, and social-life noise annoyance were all positively correlated with sleep disturbance. The correlation coefficients were 0.466 (*p* < 0.001), 0.522 (*p* < 0.001), and 0.543 (*p* < 0.001), respectively. Industrial noise annoyance was not significantly correlated with sleep disturbance (*r* = 0.060, *p* = 0.207). At the bivariate level, construction and renovation, traffic, and social-life noise showed synchronous variation with sleep disturbance, whereas industrial noise did not show a stable association.

**Table 10:** Simple correlations between source-specific noise annoyance and sleep disturbance.

Source	Spearman *r*	*p*
Industrial noise annoyance	0.060	0.207
Construction and renovation noise annoyance	0.466	<0.001
Traffic noise annoyance	0.522	<0.001
Social-life noise annoyance	0.543	<0.001

Spearman correlations are reported. Higher source-specific annoyance scores indicate greater annoyance from the corresponding noise source. *p*-values are two-sided.

#### 4.5.1 Multivariable independent associations of four noise sources

When the four source-specific noise annoyance variables entered the multivariable model simultaneously, source differences remained. The overall model was significant (*F* = 59.531, *p* < 0.001), with R^2^ = 0.684 and adjusted *R*^2^ = 0.664. Construction and renovation noise annoyance was positively associated with sleep disturbance (*p* < 0.001, 95% CI [0.267, 0.386]), traffic noise annoyance was also significant (*p* < 0.001, 95% CI [0.260, 0.382]), and social-life noise annoyance was positively associated with sleep disturbance (*p* < 0.001, 95% CI [0.334, 0.453]). Industrial noise annoyance did not reach statistical significance (*p* = 0.240, 95% CI [−0.023, 0.093]) (Table 11).

**Table 11:** Multivariable independent associations between source-specific noise annoyance and sleep disturbance.

Variable	*β*	SE (HC3)	*t*	*p*	LLCI	ULCI	Conclusion
Industrial noise annoyance	0.035	0.030	1.174	0.240	−0.023	0.093	Not supported
Construction and renovation noise annoyance	0.326	0.030	10.773	<0.001	0.267	0.386	Supported
Traffic noise annoyance	0.321	0.031	10.267	<0.001	0.260	0.382	Supported
Social-life noise annoyance	0.393	0.030	12.988	<0.001	0.334	0.453	Supported

*N* = 451, *R*^2^ = 0.684, adjusted *R*^2^ = 0.664, *F* = 59.531, model *p* < 0.001. *β* = standardized coefficient; SE = HC3 standard error; LLCI/ULCI = lower/upper limit of the 95% confidence interval. Higher source-specific annoyance scores indicate greater annoyance from the corresponding noise source.

#### 4.5.2 Coefficient-difference tests

Coefficient-difference tests further showed significant differences between industrial noise and the other three sources. The difference between industrial noise and construction and renovation noise was significant (difference = −0.292, SE = 0.040, *z* = −7.272, *p* < 0.001). The difference between industrial noise and traffic noise was significant (difference = −0.286, SE = 0.044, z = −6.460, *p* < 0.001), and the difference between industrial noise and social-life noise was also significant (difference = −0.358, SE = 0.045, *z* = −8.007, *p* < 0.001). These results indicate that the associations for construction and renovation, traffic, and social-life noise were all stronger than that for industrial noise. The differences among construction and renovation, traffic, and social-life noise were not statistically significant, so the data do not support ranking these three non-industrial sources by association strength (Table 12).

**Table 12:** Coefficient-difference tests for source-specific noise annoyance.

Coefficient difference	Difference	SE	*z*	*p*	Interpretation
Industrial - construction and renovation	−0.292	0.040	−7.272	<0.001	Construction and renovation higher than industrial
Industrial - traffic	−0.286	0.044	−6.460	<0.001	Traffic higher than industrial
Industrial - social-life	−0.358	0.045	−8.007	<0.001	Social-life higher than industrial
Construction and renovation - traffic	0.006	0.047	0.122	0.903	Not significant
Construction and renovation - social-life	−0.067	0.046	−1.463	0.143	Not significant
Traffic - social-life	−0.073	0.046	−1.594	0.111	Not significant

Difference values compare standardized coefficients between source-specific noise annoyance variables. SE, standard error. Positive values indicate a higher coefficient for the first source; negative values indicate a higher coefficient for the second source.

Overall, the exploratory question received partial support. Associations between source-specific noise annoyance and sleep disturbance were heterogeneous. Construction and renovation, traffic, and social-life noise were more stable sleep-disturbance-related sources in the present sample, while industrial noise did not show a stable association. The coefficient-difference tests showed that the three non-industrial sources had significantly stronger associations than industrial noise, but did not show significant differences among the three non-industrial sources.

## 5 Discussion

### 5.1 Main findings and relation to previous research

This study examined the relationships among noise exposure, sleep disturbance, and health behavior represented by physical activity in a sample of urban community residents. The results show that subjective noise annoyance and self-reported residential noise-environment proxy characteristics were both associated with sleep disturbance, and that sleep disturbance formed a statistical mediation pathway between noise exposure and physical activity level.

#### 5.1.1 Pathway linking noise exposure, sleep disturbance, and health behavior

The central finding is that the pathway from noise exposure to sleep disturbance can be extended to health behavior. The mediation test showed a statistical pathway from overall noise exposure to lower health behavior scores through sleep disturbance. This suggests that the relevance of noise exposure for residents’ behavior may not stop at disturbed sleep itself. It may also relate to daytime activity through impaired recovery.

This finding connects with existing noise-health research and extends it to a behavioral outcome. Previous studies have often understood the health relevance of noise exposure through annoyance, sleep disturbance, stress responses, and distal health outcomes (16, 57). The present results show that sleep disturbance is not only a proximal indicator of noise exposure, but is also associated with residents’ daytime health behavior. The public health meaning of noise exposure should therefore include whether inadequate sleep recovery is associated with residents’ capacity to maintain self-directed health behavior.

The finding also responds to sleep and health-behavior research on recovery resources and behavioral maintenance. Existing studies show that sleep status is associated with next-day activity levels and that insufficient or poor-quality sleep may weaken daytime energy, behavioral initiation, and self-regulation (39–41, 58–62). By placing this relationship in a community noise context, this study suggests that health behavior should not be interpreted only as an individual lifestyle choice. It may also be related to residential environmental stress and sleep-recovery conditions (10, 63–65). The study therefore links the noise-sleep and sleep-health behavior evidence chains and provides exploratory evidence for understanding how community noise is associated with health behavior through daily recovery.

#### 5.1.2 Source-specific noise and sleep disturbance

The source-specific analysis showed that construction and renovation, traffic, and social-life noise were more stably associated with sleep disturbance, whereas industrial noise did not show the same pattern. Because the differences among the three non-industrial sources were not statistically significant, this study does not rank them by association strength.

This result should be interpreted mainly from the perspective of sample exposure contexts and residents’ everyday experience. For ordinary urban community residents, construction and renovation, traffic, and social-life noise are more likely to occur near residential space and overlap with rest periods, home activities, and neighborhood life. By contrast, the influence of industrial noise depends more on residential location, functional zoning, and distance from the source. In a sample that is not concentrated in industrial-adjacent communities, exposure intensity and within-sample variation for industrial noise may be limited. The absence of a stable association for industrial noise in this sample therefore should not be read as evidence that industrial noise has low health risk. Results may differ in industrial-adjacent communities or occupational exposure groups.

The finding also shows the methodological value of source classification in community noise risk identification and provides empirical support for source-specific governance. Overall noise exposure can summarize residents’ general noise experience, but it may mask differences in how sources enter sleep contexts. Existing noise-health research shows that sleep disturbance is related not only to average sound levels, but also to nighttime events, temporal source patterns, residential conditions, and avoidability (29, 34). Although the present source-specific analysis is based on residents’ self-reported perceptions rather than objective sound-level data, it suggests that distinguishing noise sources in resident-side health information can help identify which sources are more likely to co-occur with sleep disturbance in concrete community life contexts.

### 5.2 Practical implications

#### 5.2.1 Extending noise governance to residents’ rest and recovery

The results suggest that noise governance should not focus only on whether noise triggers complaints. It should also consider whether noise repeatedly enters residents’ rest contexts and interferes with the practical enjoyment of rest and sleep tranquility. The association between noise exposure and sleep disturbance indicates that residents may first experience noise through difficulty falling asleep, sleep interruption, reduced recovery, and daytime fatigue. These problems may not always become formal complaints, but they may remain associated with quality of life and the maintenance of health behavior.

Community noise governance can therefore extend from single nuisance events to the time, frequency, and context of noise occurrence. Noise that repeatedly occurs at night, during lunch breaks, or near residential space may provide an important signal of impaired rest and recovery even when a single event does not reach the threshold for judicial relief. This interpretation keeps the legal claim at the level of evidentiary relevance: resident-side sleep disturbance can support risk recognition and remedy design, but it cannot by itself prove legal causation. Resident-side information should therefore be used as an early-warning and contextual layer that complements, rather than replaces, medical-service records, environmental monitoring, and objective sound-level assessment. It can help identify where monitoring and intervention may be needed before survey associations are treated as evidence of health effects.

#### 5.2.2 Connecting source-specific governance with how noise enters daily life

The exploratory source-specific analysis showed more stable associations for construction and renovation, traffic, and social-life noise, while industrial noise did not show a stable association in the current sample. This does not mean that industrial noise should be ignored. It means that source-specific governance should not rely only on source names, but should consider how each source enters residents’ life contexts.

For construction and renovation noise, relevant governance issues include timing, continuous work, and high-intensity transient noise. For traffic noise, attention should be paid to nighttime honking, roads close to residential buildings, lower-floor exposure, and insufficient window insulation. For social-life noise, relevant contexts include commercial loudspeakers, nighttime business activity, public entertainment, and neighborhood activities. Industrial-adjacent communities still require separate assessment of exposure and health risk.

#### 5.2.3 Considering sleep-recovery conditions in health behavior intervention

The mediation results show a statistical pathway from noise exposure to health behavior through sleep disturbance. Lower health behavior scores should therefore not be attributed only to weak personal intention. They may also be related to inadequate sleep recovery. Long-term noise disturbance may be associated with lower daytime energy, willingness to be active, and self-regulatory capacity, increasing the difficulty of maintaining health behavior.

When interpreting insufficient health behavior among community residents, it is therefore necessary to consider residential environmental stress and rest-recovery conditions, not only exercise habits or health awareness. For residents with substantial sleep disturbance, encouraging more activity may have limited effect unless rest environments and nighttime noise disturbance are also addressed.

### 5.3 Limitations and future research

This study has several limitations. First, the cross-sectional design cannot verify temporal ordering among noise exposure, sleep disturbance, and health behavior. The mediation results should be interpreted as statistical pathway evidence rather than causal inference. Second, noise exposure was measured through subjective annoyance and self-reported residential noise-environment proxy indicators rather than objective sound-level monitoring. The results may therefore be affected by individual perception, affective state, and residential experience. Third, the sleep disturbance measure was adapted from PSQI-related dimensions but is not equivalent to the standard PSQI total score. The health-behavior measure was also based on self-report and may involve recall bias and measurement error. Fourth, industrial noise did not show a stable association in the current sample, which may reflect exposure distribution or the limited number of industrial-noise items. This finding should not be used to deny potential risk in industrial-adjacent communities or occupational exposure groups. Fifth, the sample consisted of urban community residents, so the conclusions are most suitable for explaining associations among noise perception, sleep disturbance, and health behavior in ordinary community settings. Extrapolation to specific high-exposure communities, rural areas, or occupational settings should be cautious.

Future research can proceed in three directions. First, longitudinal designs, diary methods, or wearable-device data can be used to test temporal relationships and within-day linkages among noise exposure, sleep disturbance, and health behavior. Second, subjective noise annoyance can be combined with objective sound monitoring, noise maps, or residential spatial information to distinguish the contributions of acoustic exposure, individual perception, and residential conditions. Third, comparative studies across different urban functional zones, community types, and high-exposure settings can examine how source structures shape the association between sleep disturbance and health behavior.

## 6 Conclusion

Based on questionnaire data from 451 urban residents, this study examined the relationships among residential noise exposure, sleep disturbance, and health behavior. The results show that subjective noise annoyance and self-reported residential noise-environment proxy indicators were positively associated with sleep disturbance, and that sleep disturbance formed a statistical mediation pathway between noise exposure and health behavior scores. The exploratory source-specific analysis showed more stable associations between sleep disturbance and construction and renovation, traffic, and social-life noise, while industrial noise did not show a stable association in the current sample. These cross-sectional self-reported findings should be interpreted as resident-side statistical evidence for risk identification, not as causal evidence of exposure effects. Practically, the findings suggest that community noise governance should attend to residents’ rest and recovery conditions and that source-specific governance should consider how different noise sources enter residential space and rest periods.

## Data Availability

The raw data supporting the conclusions of this article will be made available by the authors, without undue reservation.
